# Geogenomic Segregation and Temporal Trends of Human Pathogenic *Escherichia coli* O157:H7, Washington, USA, 2005–2014^1^

**DOI:** 10.3201/eid2401.170851

**Published:** 2018-01

**Authors:** Gillian A.M. Tarr, Smriti Shringi, Amanda I. Phipps, Thomas E. Besser, Jonathan Mayer, Hanna N. Oltean, Jon Wakefield, Phillip I. Tarr, Peter Rabinowitz

**Affiliations:** University of Calgary, Calgary, Alberta, Canada (G.A.M. Tarr);; Washington State University, Pullman, Washington, USA (S. Shringi, T.E. Besser);; University of Washington, Seattle, Washington, USA (A.I. Phipps, J. Mayer, J. Wakefield, P. Rabinowitz);; Washington State Department of Health, Shoreline, Washington, USA (H.N. Oltean);; Washington University School of Medicine, St. Louis, Missouri, USA (P.I. Tarr)

**Keywords:** Shiga-toxigenic Escherichia coli, Escherichia coli O157, spatial analysis, phylogeography, bacteria, Washington, United States, food safety, lineage, Ib, IIa, IIb, geographic distribution

## Abstract

The often-noted and persistent increased incidence of *Escherichia coli* O157:H7 infections in rural areas is not well understood. We used a cohort of *E. coli* O157:H7 cases reported in Washington, USA, during 2005–2014, along with phylogenomic characterization of the infecting isolates, to identify geographic segregation of and temporal trends in specific phylogenetic lineages of *E. coli* O157:H7. Kernel estimation and generalized additive models demonstrated that pathogen lineages were spatially segregated during the period of analysis and identified a focus of segregation spanning multiple, predominantly rural, counties for each of the main clinical lineages, Ib, IIa, and IIb. These results suggest the existence of local reservoirs from which humans are infected. We also noted a secular increase in the proportion of lineage IIa and IIb isolates. Spatial segregation by phylogenetic lineage offers the potential to identify local reservoirs and intervene to prevent continued transmission.

*Escherichia coli* O157:H7 infections cause major public health challenges. Most *E. coli* O157:H7 infections occur sporadically, and the source of infection is often difficult to identify with certainty (*1**,**2*). Many reported infections are attributed to food vehicles (*1*), but studies have implicated other risk factors, and environmental transmission may be particularly notable in rural areas (*3**–**7*). Overall, the frequency of infections with *E. coli* O157:H7 has fallen in the United States, which is likely related to improved food safety (*8*), but it is not clear that rural incidence has also fallen.

Residing in a rural area confers increased risk for *E. coli* O157:H7 infection (*9**,**10*). *E. coli* O157:H7 can persist in certain locales, posing ongoing risk to humans. Multiple studies demonstrate that specific strains persist within cattle farms and spread to neighboring farms (*11**–**15*). The reservoirs enabling this persistence may include water, soil, and wild birds (*16**–**19*). It is, therefore, possible that humans incidentally acquire *E. coli* O157:H7 infections because they reside in a geographic region with a persistent reservoir. Using a generalizable population-based cohort, we sought to test the hypothesis that there are geographic foci of related *E. coli* O157:H7 infections, most likely of environmental origin, taking into account the genomic relatedness of different isolates (*20**,**21*) and the geographic, temporal, and secular attributes of their corresponding infections.

## Methods

### Study Population and Pathogen Characterization

We conducted a population-based retrospective cohort study of all culture-confirmed *E. coli* O157:H7 cases reported to the Washington State Department of Health (DOH; Shoreline, WA, USA) during 2005–2014. *E. coli* O157:H7 case reporting mandated by the Washington Administrative Code occurs primarily through diagnostic laboratories and healthcare providers. Local health jurisdictions use a standardized DOH case report form to abstract medical records; interview case-patients to obtain demographic information (including residence address), potential exposures, and details of the course of illness; and determine the most likely source of infection. For this study, case addresses were geocoded and census block groups determined. Case data were deidentified for analysis. This study was deemed exempt by the Washington State Institutional Review Board.

All *E. coli* O157:H7 isolates are sent to DOH for microbiologic confirmation and XbaI pulsed-field gel electrophoresis (PFGE) typing. We obtained isolates from DOH and determined their lineage according to the phylogenetic tree developed by Bono et al. (*20*) and expanded by Jung et al., who identified some lineages as clinical and others as bovine-biased (*21*). We used the Jung et al. 48-plex single-nucleotide polymorphism (SNP) assay to type a subset of isolates (*21*). We assumed that all isolates with a given PFGE pattern would be SNP typed to the same lineage. Thus, we typed >1 isolate from each PFGE pattern in the dataset and inferred the lineage of nontyped isolates. Concordance among isolates with identical PFGE profiles was confirmed (Technical Appendix 1). We analyzed the clinically common lineages Ib, IIa, and IIb separately and analyzed the bovine-biased and remaining sparsely represented lineages (*21*) as a clinically rare group. (Genomic data, with limited metadata, on all isolates used in the study are provided in Technical Appendix 2)

### Phylogenetic Lineage Spatial Segregation

Spatial segregation is the ecologic concept that one species or species type is more likely to be surrounded by like than by nonlike individuals (*22*). We used Diggle’s kernel estimation method (*23*) and spatialkernel package (*24*) in R (*25*) to test spatial segregation of *E. coli* O157:H7 by phylogenetic lineage (online Technical Appendix). In brief, we first estimated a smoothed probability surface for each lineage by comparing the distance between cases infected with the same lineage to the distance between cases infected with different lineages. A peak in the lineage-specific probability surface indicates an area with a high probability of that lineage, relative to the distribution of the other lineages. For example, if 80% of cases in a given proximity are infected with lineage Ib but in all other areas lineage Ib causes only 50% of cases, we would observe a peak in the lineage Ib-specific probability surface, suggesting spatial segregation. To determine overall spatial segregation, the probability surfaces were compared with a null distribution in which the proportion of infections caused by each lineage is constant across space. 

We next sought to account for potential confounders and to detect geographic trends. To do so, we modeled the risk surface using a multinomial generalized additive model (GAM). We estimated the effect of a bivariate thin plate regression spline smooth of latitude and longitude on the odds of infection with a given lineage compared with the most common lineage. This smoothing technique produces a risk surface that can vary flexibly across both horizontal and vertical coordinates. In this analysis, we compared lineages IIa and IIb and the group of clinically rare lineages separately with lineage Ib, which served as the reference (most common) lineage. The model was adjusted for sex and age group (<5, 5–9, 10–19, 20–59, and >60 years); isolates from cases of unknown age (n = 1) or sex (n = 10) were excluded from analysis. We estimated parameters using restricted maximum likelihood and the mgcv package in R (*26**,**27*). We further conducted a series of sensitivity analyses to determine the robustness of our results by seeking to confirm our results with 2 independent methods: Dixon’s nearest-neighbor test (*22*) and multinomial spatial scan statistics (*28*) (Technical Appendix 1).

### Temporal Variation in Spatial Segregation

To determine whether spatial segregation of lineages varied over time, we replicated our spatial segregation analyses incorporating time. To do so, we split the years of analysis into 3 intervals (2005–2007, 2008–2010, and 2011–2014) and calculated a kernel-based estimate of spatial segregation for each. We evaluated the effect of time in the multinomial GAM by adding year to the model as a continuous variable, testing the effect of year as both a linear term and as a smoothed term using a thin plate spline. The thin plate spline allows the association between lineage and year to smoothly change in magnitude and direction.

### Exploratory Risk Factor Analysis

We explored potential drivers of segregation by testing the association of risk factors included on the DOH case report form with each lineage compared with the reference lineage Ib. Using multinomial GAMs adjusting for sex, age, year, and latitude and longitude as a thin plate spline bivariate smoother, we tested each risk factor (online Technical Appendix Table 1). In addition to the statewide analyses, region-specific analyses were conducted for the 3 regions with the highest *E. coli* O157:H7 incidence to determine locally key associations. Regions were defined based on major population centers, areas of increased agricultural intensity, and observed segregation clusters, and models were adjusted for sex, age, and year.

## Results

**Figure 1:**
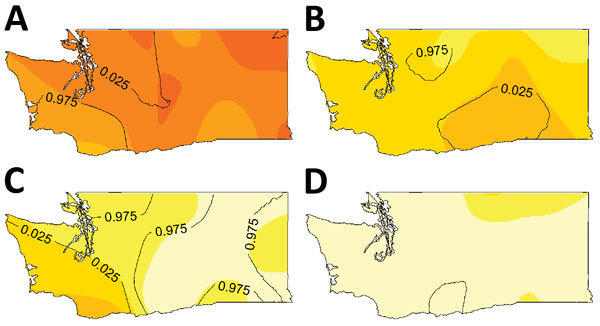
*Escherichia coli* O157:H7 lineage frequency among culture-confirmed human cases reported in Washington, USA, 2005–2014. A) Lineage Ib; B) lineage IIa; C) lineage IIb; D) rare lineages (12 different clinically rare lineages). Lineage-specific probability surfaces were determined by kernel-based estimation of spatial segregation. Darker shading indicates higher risk for that lineage. Contour lines marked 0.025 define areas in which there is a high probability of cases being caused by a given lineage, suggesting spatial segregation. Contour lines marked 0.975 define areas in which there is a low probability of cases being caused by the given lineage.

During the study period, 1,160 *E. coli* O157:H7 cases were reported to DOH. Of these, 33 isolates, representing 31 PFGE types, were not available for typing (Technical Appendix 1), and isolates from 6 cases were excluded as biochemically atypical *E. coli* O157:H7 (Technical Appendix 1 Figure 1). We SNP typed 793 isolates and, by extension, matched another 328 to a known lineage using PFGE, enabling us to assign a specific lineage of *E. coli* O157:H7 to isolates from 1,121 cases. Ten cases lacked address data and were excluded, leaving 1,111 cases for analysis.

Lineages Ib, IIa, and IIb, in descending order, were the most common lineages (Table). Twelve clinically rare lineages were identified, including 2 not previously described, encompassing 45 unique PFGE types (Technical Appendix 1 Figure 1). Lineage Ib comprised 210 PFGE types, whereas lineage IIa comprised only 38 PFGE types and lineage IIb 26 PFGE types (Technical Appendix 1 Figure 1). Lineage IIa contained an average of 7 (SD 14) and IIb an average of 8 (SD 25) isolates per PFGE type, compared with 3 (SD 5) for lineage Ib and 1 (SD 2) for the clinically rare lineages (Table).

**Table:** *Escherichia coli* O157:H7 lineage frequency by case characteristic among culture-confirmed human cases reported in Washington, USA, 2005–2014*

Variable	Lineage Ib	Lineage IIa	Lineage IIb	Rare lineages†
Total	586 (52.7)	260 (23.4)	199 (17.9)	66 (5.9)
Mean isolates per PFGE type (SD)‡	2.8 (5.3)	6.8 (14.3)	7.7 (24.7)	1.5 (1.7)
Sex				
F	333 (56.8)	163 (62.7)	105 (52.8)	33 (50.0)
M	244 (41.6)	97 (37.3)	94 (47.2)	32 (48.5)
Unknown	9 (1.5)	0	0	1 (1.5)
Age group, y				
<5	119 (20.3)	72 (27.7)	63 (31.7)	10 (15.2)
5–9	81 (13.8)	32 (12.3)	33 (16.6)	12 (18.2)
10–19	97 (16.6)	51 (19.6)	31 (15.6)	6 (9.1)
20–59	207 (35.3)	81 (31.2)	49 (24.6)	29 (43.9)
≥60	81 (13.8)	24 (9.2)	23 (11.6)	9 (13.6)
Unknown	1 (0.2)	0	0	0
HUS				
Yes	37 (6.3)	18 (6.9)	20 (10.0)	0
No	526 (89.2)	236 (90.1)	173 (86.1)	67 (98.5)
Unknown	27 (4.6)	8 (3.1)	8 (4.0)	1 (1.5)

*Values are no. (%) except as indicated. HUS, hemolytic uremic syndrome; PFGE, pulsed-field gel electrophoresis. †Twelve clinically rare lineages. ‡PFGE type percentages indicate the proportion of PFGE types with an assigned lineage (n = 355) belonging to each lineage.

Distribution of cases by sex, age group, and hemolytic uremic syndrome (HUS) status varied by lineage (Table). Lineage IIa and IIb isolates originated disproportionately from children <5 years of age compared with isolates in lineage Ib. Patients infected with lineage IIb bacteria also had higher frequencies of HUS (10%) than other patients (6%). None of the patients with infections caused by isolates from the clinically rare lineages developed HUS.

### Spatial Segregation

The result of Diggle’s kernel estimation test was statistically significant (p = 0.001), suggesting spatial segregation. Lineage-specific probability surfaces showed separate, distinct peaks for lineages Ib, IIa, and IIb (Figure 1). The southwest region of Washington was marked by segregation of lineage IIb isolates and correspondingly lower probability of isolating lineage Ib from cases. Spatial segregation was observed for lineage Ib isolates in northwest Washington and for lineage IIa isolates in the south-central region. There was low probability of lineage IIb isolates in both these areas. Sensitivity analysis corroborated these results (online Technical Appendix).

**Figure 2:**
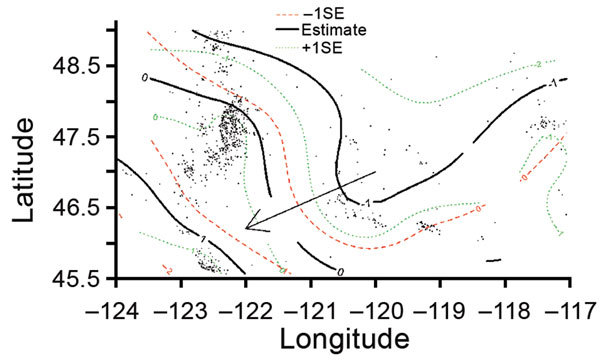
Risk surface of *Escherichia coli* O157:H7 lineage IIb relative to lineage Ib using a multinomial generalized additive model and a bivariate thin plate smooth function for longitude and latitude for culture-confirmed human cases reported in Washington, USA, 2005–2014. The black contour lines show the mean effect estimate for lineage IIb relative to Ib as latitude and longitude change. The 0-marked black line indicates no effect. The 1-marked black line indicates greater proportional incidence of lineage IIb toward the southwest corner of the area as compared to lineage Ib (p<0.001). The arrow indicates the general direction of the trend from higher Ib risk to higher IIb risk. Dashed red lines show the effect estimate 1 standard error (SE) below (to the south and west) the mean estimate. Dotted green lines show the effect estimate 1 standard error above (to the north and east) the mean estimate.

Consistent with the kernel regression results, the adjusted GAM risk surface of lineage IIb varied significantly from that of Ib (p<0.001), providing additional support of the spatial segregation. The frequency of lineage IIb isolation was greater than the frequency of Ib in the southwest region, but this imbalance diminished as latitude and longitude increased (Figure 2), that is, in areas northward and eastward. This spatial pattern was also observed in the kernel estimation map of lineage IIb (Figure 1). The risk surfaces of lineage IIa and the clinically rare lineage group did not differ significantly from that of Ib (online Technical Appendix Table 2). In sensitivity analyses designed to gauge the robustness of results to model assumptions, the spatial risk surface of lineage IIb consistently varied significantly from the risk surface of lineage Ib (Technical Appendix 1 Table 2). The spatial risk surface of lineage IIa also varied significantly from the risk surface of lineage Ib in some sensitivity analyses, similar to the spatial distribution in the kernel estimation lineage IIa–specific probability surface.

We also found significant differences in lineage by age of infected patients, independent of geography. The likelihood of being an adult (age ranges 20–59 and >60 years of age) versus being a toddler (<5 years of age) was lower among IIa-infected patients than among Ib-infected patients (20–59 years odds ratio [OR] 0.65, 95% CI 0.44–0.96; >60 years OR 0.49, 95% CI 0.28–0.85). The odds of being 20–59 years of age versus <5 years were also lower among IIb-infected patients than among Ib-infected patients (OR 0.44, 95% CI 0.28–0.69). Thus, adults comprised a smaller proportion of patients infected with lineage IIa or IIb *E. coli* O157:H7 than of those infected with lineage Ib. We found no significant differences by sex.

### Temporal Variation

**Figure 3:**
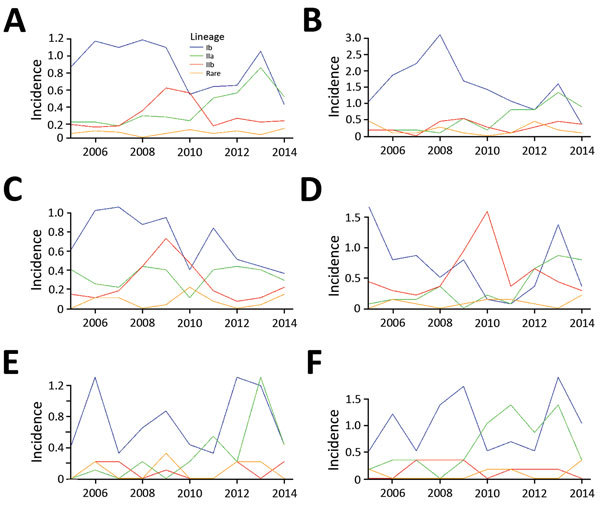
Annual incidence (per 100,000 population) of reported *Escherichia coli* O157:H7 cases by phylogenetic lineage, Washington, USA, 2005–2014. A) Statewide; B) northwest region; C) Seattle–Tacoma region; D) southwest region; E) northeast region; F) south-central region. Regions were defined according to major demographic characteristics and patterns of segregation observed in analyses for the whole period. The northwest region experienced the highest peak incidence. The Seattle–Tacoma region and the northeast region experienced the lowest incidences. “Rare” indicates 12 different clinically rare lineages.

The incidence of *E. coli* O157:H7 averaged 1.73/100,000 population during the study period. Although incidence fluctuated from a low of 1.37/100,000 population in 2014 to a maximum of 2.28/100,000 population in 2013, we found no discernible trend in overall incidence. However, the composition of the *E. coli* O157:H7 population shifted over time (Figure 3). In the GAM analysis including year as a linear term, incidence relative to lineage Ib increased over time for lineage IIa (OR 1.26, 95% CI 1.19–1.34), lineage IIb (OR 1.10, 95% CI 1.03–1.17), and clinically rare lineages (OR 1.13, 95% CI 1.02–1.26).

We observed a peak of lineage IIb incidence during the middle of the study period in southwest Washington and the Seattle–Tacoma region (Figure 3). Using kernel regression, we identified statistically significant temporal variation in spatial segregation across intervals (p = 0.001). We observed statistically significant overall spatial segregation only during the 2008–2010 interval (p = 0.001). Some portion of the southwest region of the state showed increased probability of lineage IIb isolation during all intervals, and lineages Ib and IIa were segregated during 2008–2010 and 2011–2014 (Video). Cross-validated log-likelihood bandwidths used in these analyses ranged from 0.73 to 1.0. In sensitivity analysis, a lower bandwidth yielded statistically significant spatial segregation during all periods (Technical Appendix 1). Latitude and longitude remained significant predictors of Ib in GAMs that included year (Technical Appendix 1 Table 2).

**Video:**
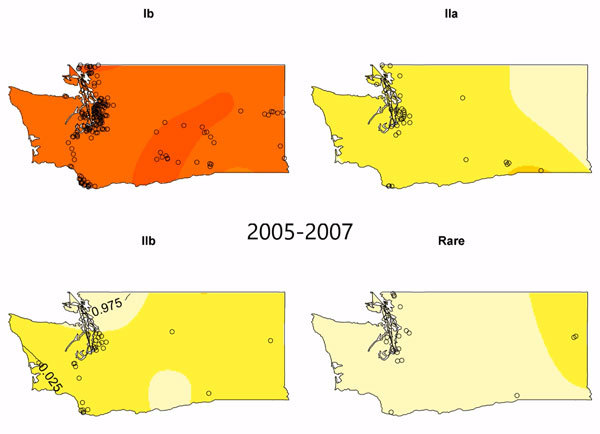
Lineage-specific probability surfaces for *Escherichia coli* O157:H7 from culture-confirmed human cases reported in Washington, USA, 2005–2014. Probabilities were determined by kernel-based estimation of spatial segregation for 3 intervals: 2005–2007 (n = 305, bandwidth = 1.0000); 2008–2010 (n = 367, bandwidth = 0.7256); and 2011–2014 (n = 439, bandwidth = 0.9314). Overall spatial segregation was not statistically significant for the 2005–2007 interval (p = 0.769) or 2011–2014 interval (p = 0.138) but was statistically significant for the 2008–2010 interval (p = 0.001). Circles indicate case locations. Darker hues indicate higher risk. Contour lines marked 0.025 define areas in which there is a high probability of cases being caused by a given lineage, suggesting spatial segregation. There is an area of statistically significant spatial segregation for lineage IIb in all 3 intervals. Contour lines marked 0.975 define areas in which there is a low probability of cases being caused by the given lineage.

### Sensitivity Analysis

Alternate analytic approaches confirmed the results of our primary analyses. Dixon’s test for spatial segregation identified statistically significant spatial segregation overall, as well as for lineages Ib, IIa, and IIb (Technical Appendix 1 Tables 3, 4). Three clusters identified using multinomial spatial scan statistics paralleled areas of segregation found in the kernel regression analysis and were consistent with the southwest trend toward proportionally greater IIb observed in the multinomial GAM (Technical Appendix 1 Figures 3, 4).

To focus on potential local reservoirs, which are not likely to be human, we also conducted the analysis without cases due to presumptive person-to-person transmission (Technical Appendix 1). We used the most likely source of infection documented on the DOH case report form to exclude patients most likely infected by other persons. After discounting secondary transmission, we observed spatial segregation using the kernel estimation method (p = 0.002). The risk surface of lineage IIb still varied significantly from that of Ib (p<0.001). The trend toward greater IIb relative to Ib risk in southwest Washington was consistent with the analysis of all cases, but relative IIb risk was substantially lower in the northeast region than that observed in the primary analysis. This pattern suggests that lineage IIb infections in northeast, but not southwest, Washington may be disproportionately attributed to secondary transmission compared with Ib infections. Finally, we found no evidence of case ascertainment bias that could independently explain our results (Technical Appendix 1).

### Exploratory Risk Factor Analysis

Statewide, patients infected with lineage IIa *E. coli* O157:H7 were more likely to have reported raw fruit or vegetable consumption than those infected with lineage Ib pathogens (OR 1.81, 95% CI 1.05–3.11). Patients infected with lineage IIb *E. coli* O157:H7 were more likely to have reported raw milk consumption than those infected with lineage Ib pathogens (OR 2.46, 95% CI 1.15–5.28). All examined risk factors and associations are summarized in Technical Appendix 1 Table 1.

## Discussion

The geographic differences and temporal trends in the relative frequencies of lineages of *E. coli* O157:H7 from cases in Washington demonstrate that, in addition to genomic variation reported at the national level (*29**,**30*), persistent geogenomic variation exists at the regional level. Several geospatial associations warrant elaboration. In all analyses, lineage IIb cases were segregated in the southwest region of the state. Southwest Washington includes Olympia, the state capital, as well as suburbs of Portland, Oregon, north of the Columbia River; however 27% of the population in the 12 southwest region counties is considered rural, compared with 16% of the state as a whole (*31*). Small farms are common. The southwest region is home to >20% of the state’s farms but accounts for only 7.1% of its cattle and 6.3% of farm acreage (*32*). Roosevelt elk roam the southwest region, and elk elsewhere in the country have been identified as Shiga toxin–producing *E. coli* carriers (*33*). Water is also a potential factor in *E. coli* O157:H7 epidemiology in the southwest region, which has abundant coastal and river exposures. The largest recognized IIb outbreak in this region accounted for only 11 cases linked to a particular daycare center (out of 77 IIb infections in the region), so the observed segregation is unlikely due to a single point source. Notably, lineages IIa and IIb have the greatest overlap with the putatively hypervirulent clade 8 (*34*), making their segregation of particular concern.

Lineage IIb isolates were relatively uncommon in the northwest and south-central regions of Washington, both major cattle-production regions. Lineage Ib showed segregation in the northwest and IIa in the south-central region in some analyses, although their adjusted risk surfaces did not differ significantly, suggesting overlap. More research is needed to clarify why lineage IIb has not yet also established itself in areas with abundant cattle.

The presence of spatially segregated lineages indicates local environmental reservoirs producing infections above and beyond those caused by widely distributed exogenous sources such as food. We propose that persistent spatial segregation of a lineage could reflect a founder effect, in which an ancestral pathogen has become established in a region, persisted, and expanded and occasionally crosses into the human population. Such a dynamic would result in phylogenetically similar bacteria being isolated in the same general geographic region separated by months or years, as we have observed in this study. A possible precedent exists in a report of 2 cases from Webster County, Missouri, USA (*35*). Our findings are also consistent with those of Jaros et al., who found that geography explains some variation in *E. coli* O157:H7 strains in New Zealand (*36*). In addition, prior work from Washington demonstrated shifts over time in the Shiga toxin genotypes of *E. coli* O157:H7 (*37*).

The clinical infections in our study were dominated by *E. coli* O157:H7 in lineages Ib, IIa, and IIb, consistent with the results of Jung et al. (*21*). Our work is also consistent with a national study showing that lineage Ib *E. coli* O157:H7 causes most clinical cases in the United States (*30*). Relative to lineage Ib, Washington experienced statistically significant increases in the other clinically common lineages during the study period. The increase is most dramatic for lineage IIa, which appears to have emerged in most regions in the latter half of the study period (Figure 3). This difference could reflect the changing epidemiology of *E. coli* O157:H7 discussed by Rivas et al., owing to changes in food sources and consumption, or, possibly, pathogen evolution (*38*). Lineage IIa *E. coli* O157:H7 has emerged as a major cause of disease across the state, suggesting a disseminated driver of infections for this lineage overall. Lineage IIa’s observed association with raw fruit and vegetable consumption, as compared with that for lineage Ib, is consistent with this hypothesis. The south-central region of Washington, identified in some analyses as an area of IIa segregation, experienced an uptick in IIa infections earlier than in other regions. This area includes the Yakima Valley, an area of higher agricultural intensity; a local IIa reservoir in this region could produce the observed segregation independent of statewide trends.

Our findings suggest exposures that may be preferentially associated with particular lineages. Specifically, we observed associations of lineage IIb with drinking untreated/unchlorinated water and raw milk in the southwest region, where this lineage is segregated (Technical Appendix 1 Table 1). There may be a lineage IIb reservoir in animals producing raw milk in this area, or bacteria from environmental reservoirs in the area may spill over into these animals and local water sources. Only 1 small, recognized raw milk outbreak in 2005 was noted on the DOH case report forms, making it unlikely that a single source is responsible for the association we found over time. It is possible that some *E. coli* O157:H7 lineages may be especially successful in surviving in particular vehicles or environments, such as raw produce or unpasteurized milk or water. Secular changes might also be the result of shifting environmental exposure risk if, for example, contact between a reservoir and humans varies over time. Better knowledge of small-intermediate area transmission patterns will open opportunities for intervention if reservoirs can be identified.

Our study is limited by its reliance on SNP data to define phylogenetic lineages. Whole-genome sequencing would have supported finer resolution of relatedness, particularly among isolates that were segregated in time and space, and enabled us to trace the history of segregated clusters. Such an analysis would not necessarily alter our conclusions, however, because evolution of specific clades of *E. coli* O157:H7 within a region, and the identification of different sublineages, would still be consistent with a founder effect. In fact, the precise delineation of the chromosomal architecture in these pathogens might actually confirm a common progenitor, as demonstrated from worldwide analyses of *E. coli* O157:H7 (*39*). Our use of phylogenetic lineages rather than PFGE profiles is also a strength of the work, because PFGE does not put differences into evolutionary perspective (*39*). By basing the analysis on phylogenetic lineages, we captured relatedness among strains and indicate the level of *E. coli* O157:H7 diversity as it circulates through its host populations. We also used multiple analytic techniques to provide confidence that our results were not due to assumptions made by any particular method.

In summary, clusters of spatial segregation by phylogenetic lineage in Washington suggest local reservoirs that perennially cause human disease. Further exploration of land use, human movements, and social–behavioral factors could elucidate within-region drivers of spatial segregation. We see comparison of lineage-specific spatial patterns with distributions of these and other factors as an essential next step in understanding *E. coli* O157:H7 spatial segregation. Environmental risk assessment and longitudinal studies based on our findings would also provide valuable information by identifying pathogen reservoirs that have not been identified by traditional public health surveillance and that could be mitigated by public health or environmental measures. The makeup of the *E. coli* O157:H7 population in the state is also shifting. To manage emerging lineages, attention is needed to the heterogeneity in risk factors across the phylogenetic tree. Greater knowledge of the most likely sources of infection for particular lineages has the potential to focus both outbreak investigations and efforts to identify persistent reservoirs.

Technical Appendix 1 Description of supplementary methods and analyses associated with *Escherichia coli* O157:H7 phylogenetic classification, Washington, USA, 2005–2014.

Technical Appendix 2 Genomic data on all 1,160 *Escherichia coli* O157:H7 isolates from reported, culture-confirmed cases in Washington, USA, 2005–2014.
