# Supplementary material for: Geogenomic Segregation and Temporal Trends of Human Pathogenic Escherichia coli O157:H7, Washington, USA, 2005–2014
Source: Emerg Infect Dis. 2018 Jan;24(1):32–9. doi: 10.3201/eid2401.170851 (PMC5749469; doi:10.3201/eid2401.170851)
Supplement: Technical Appendix 1 — Description of supplementary methods and analyses associated with Escherichia coli O157:H7 phylogenetic classification, Washington, USA, 2005–2014. [file 17-0851-Techapp-s1.pdf]

Article DOI: http://dx.doi.org/10.3201/eid2401.1710851

Geogenomic Segregation and Temporal Trends of Human Pathogenic *Escherichia coli* O157:H7

Technical Appendix 1

Supplementary Methods and Analyses

Assigning Phylogenetic Lineage to Non–SNP-Typed Isolates

In previous studies analyzing patterns associated with *E. coli* O157:H7 phylogenetic classification, it has been common to use a single representative isolate from each PFGE subtype (*1*–*3*). This practice masks the variability among isolates with the same PFGE fingerprint (e.g., variability in demographics, location). Further, estimation of effects at the population level is compromised, because the isolates being analyzed are not reflective of the *E. coli* O157:H7 case population distribution. To accurately make inference at the population level, we sought to include all reported cases during the study period. Because we did not have sufficient resources to SNP-type all isolates, we leveraged the assumption inherent in the single-representative-isolate approach, although not generally made explicit: isolates with the same PFGE fingerprint belong to the same phylogenetic grouping.

Our sample contained 1,160 isolates reflecting 355 unique XbaI PFGE patterns (Technical Appendix Figure 1). We SNP-typed 793 of these isolates, covering 319 PFGE subtypes. The 36 PFGE subtypes not SNP-typed were either biochemically atypical or they were not present in the isolate bank. Atypical isolates were exclusively from 2013 and 2014, the last 2 years of sampling. Missing isolates were predominantly (82%) from 2005 and 2006, the first 2 years of sampling. Of the 793 SNP-typed isolates, 570 belonged to a PFGE subtype with multiple SNP-typed isolates. Among these 570, we examined which phylogenetic lineages the isolates had been assigned via SNP typing. All but 1 PFGE subtype were assigned a consistent lineage. The one variable PFGE subtype was EXHX01.0047. It encompassed 82 isolates: 21 were not typed, 59 were typed to lineage IIa, and 2 were typed to lineage Ib. In other words, only

2 of 570 isolates (0.4%) showed aberrant lineage assignment. With this, we felt that the assumption that isolates of the same PFGE subtype would be in the same lineage held adequately well to use the SNP-typing results to assign lineage to non–SNP-typed isolates. We were able to assign lineage to 328 additional isolates by using this approach.

Spatial Segregation by Diggle's Kernel Estimation Method

Diggle's kernel estimation provides smoothed estimates of spatial segregation that take into account multiple neighbors of each case. It provides an overall test of spatial segregation and identifies statistically significant regions in the lineage-specific probability surfaces. Diggle's method assumes an underlying Poisson point process for each phylogenetic lineage. The degree of smoothing is dependent on the choice of a bandwidth. A cross-validated log-likelihood function can be used to calculate the bandwidth (*4*). We tested bandwidths between 0.02 and 1 degrees at 0.0098-degree increments to identify and then select for analysis the bandwidth (0.6472 degrees) associated with the greatest cross-validated log-likelihood. Using the selected bandwidth, we determined the lineage-specific probabilities based on the surrounding cases for each case location and plotted the lineage-specific probability surfaces on individual maps. We then calculated a test statistic for spatial segregation by summing the square of the difference between the kernel regression-estimated lineage-specific probability at a given location and the overall probability that a case isolate belongs to that lineage over all lineages and all case locations. To determine statistical significance, we performed 999 Monte Carlo replications with lineage randomly relabeled at each case location, maintaining the observed number of cases of each lineage. The proportion of test statistics greater than that observed from the data was the p-value. The analysis was conducted in R (*5*) using the spatialkernel package (*6*).

The bandwidth selected for the main analysis was used for all lineages within a given analysis. To identify the sensitivity of the kernel estimation results to the bandwidth of 0.6472 degrees that was selected, alternate bandwidths were tested: 0.02, 0.1, 0.2, 0.4, and 0.9. All yielded $p = 0.001$ for the overall test for spatial segregation. The segregation maps for individual lineages grew predictably smoother as the bandwidth was increased and identified statistically significant areas of segregation consistent with the primary result from a bandwidth of 0.6472.

Temporal variation in segregation was tested across 3 intervals: 2005–2007, 2008–2010, and 2011–2014. The slightly longer last interval is not expected to affect the validity of the

results. However, because of the greater number of cases in this interval, greater precision is expected. We calculated a new bandwidth for each new analysis and subset of the data using the cross-validated log-likelihood function. For the overall test of variation of spatial segregation across time intervals using the kernel regression method, we chose a bandwidth of 0.8236 degrees. The bandwidths chosen for each of the individual intervals were 1.0000 for 2005–2007, 0.7256 for 2008–2010, and 0.9314 for 2011–2014. Not unexpectedly, given the high degree of smoothing in the first and last periods, only the middle period had detectable overall spatial segregation ($p = 0.001$). However, all periods displayed some statistically significant spatial segregation for individual lineages (Technical Appendix Video). A bandwidth of 0.4 was also tested for each of the intervals, resulting in statistically significant tests for overall spatial segregation in each interval (2005–2007 $p = 0.037$, 2008–2010 $p = 0.001$, 2011–2014 $p = 0.014$).

Multinomial Generalized Additive Model

The multinomial GAM provides a smoothed risk surface relative to Ib, the most common lineage. Unlike the direct measures of spatial segregation, the GAM captures spatial trends without selecting a specific distance or number of neighbors across which to smooth. It does this through a flexible spline function. The GAM also supports adjustment for covariates, providing some assurance that the associations observed are not due to factors such as the distribution of cases by age. The multinomial analysis entailed logistic-type equations for each of the 3 lineage comparisons. Results of the GAM multinomial models must be interpreted conditional on having a reported *E. coli* O157:H7 illness. As such, odds ratios presented estimate risk proportional to that in the most common lineage, Ib.

We tested multiple aspects of the GAM specification. Latitude and longitude were specified individually and jointly to allow interaction. The basis dimension of the penalized regression smoother was altered to improve the effective degrees of freedom. Age and sex covariates were removed, and the form of the spline smoother was altered. Lineage IIa was used as the comparison lineage. These sensitivity analyses are summarized in Technical Appendix Table 2. None of the model perturbations meaningfully changed the primary model results. In the set of GAMs incorporating year, a trivariate smooth of latitude, longitude, and year was also tested and found to be statistically significant for lineages IIa and IIb (Technical Appendix Table 2).

Spatial Segregation by Dixon's Nearest-Neighbor Method

Another measure of spatial segregation, Dixon's nearest-neighbor method, considers only the closest neighbor of each case. It conducts no smoothing and can be expected to be sensitive to clustered outbreaks. This method does not indicate areas in which spatial segregation exists but does provide an overall test of spatial segregation, as well as for segregation of individual lineages and pairwise segregation tests. We created a 4 × 4 contingency table of nearest-neighbor counts for each lineage group. A χ^2 test with 12 degrees of freedom was used to test overall spatial segregation, and segregation was tested for each individual lineage group (Technical Appendix Table 3). We calculated Dixon's segregation index for each nearest-neighbor combination (e.g., from Ib to IIa; Technical Appendix Table 4). Dixon's pairwise segregation index is defined as:

$$S_{ij} = \log \frac{N_{ij}/(N_i - N_{ij})}{\mathrm{E}N_{ij}/(N_i - \mathrm{E}N_{ij})} = \log \frac{N_{ij}/(N_i - N_{ij})}{N_i/(N - N_j - 1)}$$

where *i* and *j* in this analysis are phylogenetic lineages (*7*). A positive value of *S* indicates association, and a negative value indicates segregation. We calculated Z-scores for each combination by comparing the observed nearest-neighbor count in each cell to the expected count. We calculated a p-value based on the Z-scores assuming an asymptotic normal distribution. We used the Dixon R package for this analysis (*8*).

We used Dixon χ^2 tests for segregation to indicate statistically significant segregation overall ($p < 0.001$) and for lineages Ib ($p = 0.046$), IIa ($p = 0.002$), and IIb ($p < 0.001$), but not for the group of clinically rare lineages (Technical Appendix Table 3). This is consistent with the findings of the kernel estimation method, which found statistically significant overall spatial segregation and identified areas of segregation for lineages Ib, IIa, and IIb. Dixon's method also tests associations between individual lineages. Pairwise nearest-neighbor comparisons showed statistically significant positive association from each of lineages Ib, IIa, and IIb to itself. Segregation was observed from Ib to IIa, IIa to the rare lineages, IIb to all other lineages, and the rare lineages to Ib (Technical Appendix Table 4).

We examined spatial segregation with Dixon's method for the 3 intervals analyzed with the kernel estimation method. Spatial segregation was found to be statistically significant with $p < 0.001$ during all 3 periods, contrasting with Diggle's method, which identified statistically

significant overall segregation only during the 2008–2010 period. However, the 2 spatial segregation tests were consistent in identifying spatial segregation of lineage IIb during all intervals ($p < 0.001$ for Dixon's method during all intervals). Additionally, Dixon's method identified segregation of lineage IIa during the 2005–2007 period ($p < 0.001$) and segregation of lineage Ib during the 2008–2010 ($p < 0.001$) and 2011–2014 ($p = 0.005$) periods.

Multinomial Spatial Scan Statistics

We used multinomial spatial scan statistics (*9*) in SaTScan (*10*) to identify clusters within which the distribution of lineages differed significantly from the distribution of lineages outside the cluster. The spatial scan statistics are designed to identify clusters of disease. In the multinomial framework used here, the clusters reflect areas within which the distribution of cases by lineage is skewed compared with the area outside the cluster. These are similar to the areas of segregation identified by the kernel regression method. However, the scan statistics look at the distribution of all 4 lineages simultaneously and not individually, thus allowing detection of clusters in which multiple lineages may be out of proportion. Like the multinomial GAM models, the multinomial spatial scan statistics must be interpreted conditionally on having a reported *E. coli* O157:H7 illness.

For the primary spatial scan statistic model, we used a maximum cluster size of 20% of cases. Statistical significance of the clusters was determined based on Monte Carlo replications under the null. Relative risks presented estimate risk of one's infection being from the given lineage inside the cluster compared with the risk outside that cluster.

We identified 3 statistically significant clusters in which the distribution of cases by phylogenetic lineage varied from the distribution in the rest of the state (Technical Appendix Figure 2). The first cluster ($p = 0.001$) contained 203 cases, was centered in the southwest region of the state, and was characterized by a higher proportion of lineage IIb cases than observed elsewhere in the state (relative risk [RR] 2.59). The second cluster ($p = 0.001$), encompassing the sparsely populated northern reaches of the state, contained 185 cases and had somewhat more Ib (RR 1.37) and rare lineage (RR 1.88) cases and fewer IIb cases (RR 0.29). The final significant cluster ($p = 0.006$) contained 79 cases in the south-central region of the state; lineage IIa was more common than elsewhere in the state (RR 1.70), IIb was uncommon (RR 0.13), and cases due to rare lineages were nearly absent (RR 0). The first cluster, dominated by IIb, and third

cluster, dominated by IIa, recapitulate the results of the kernel estimation maps and, for IIb, the GAM-generated risk surface. The second cluster, dominated by lineage Ib, is larger and centered somewhat further east than the area of segregation identified for Ib by the kernel estimation method, though still similar.

Altering the parameters of the analysis to allow lower or higher percentages of the cases to be included in clusters did not meaningfully affect the position of the clusters identified. We tested allowing clusters up to 50% of cases and 10% of cases. From the former, the main IIb-dominant and Ib/rare-dominant clusters were identified, but the IIa-dominated cluster was not. Limiting clusters to 10% of cases, all 3 clusters identified in the primary analysis were identified but with smaller numbers of included cases.

We detected variant clusters using multinomial spatiotemporal scan statistics, using year as the time scale and allowing up to 50% of the study period in a cluster, as well as purely spatial clusters. We identified 3 statistically significant clusters (Technical Appendix Figure 3). The first ($p = 0.001$) contained 76 cases reported during 2009–2012 in the southwest region of the state and had an elevated risk of lineage IIb (RR 4.45). The second cluster ($p = 0.001$) included 107 cases across the northeast region during 2005–2009. The Ib (RR 1.61) and rare (RR 1.88) lineages were elevated. The third cluster ($p = 0.002$) included only 46 cases reported during 2009–2010, with a predominance of lineage IIb (RR 3.63) and near-absence of IIa (RR 0.09). This cluster included part of Seattle, Washington's largest urban area, and areas immediately south and east.

Secondary Cases

To separate the effect of person-to-person transmission from other potential environmental factors that may result in segregation, we conducted sensitivity analyses after excluding known secondary cases. To be excluded, the most likely source of the infection had to have been identified during the public health investigation as person-to-person, or the notes had to indicate that another individual in the household or childcare situation had previously received such a diagnosis. Based on these criteria, 82 secondary cases were excluded. No meaningful changes in the results were observed. The overall test of spatial segregation was statistically significant using the kernel estimation method ($p = 0.002$) and the nearest-neighbor method ($p < 0.001$). The latitude/longitude smooth of lineage IIb from the multinomial GAM is statistically

significantly different from that of lineage Ib ($p < 0.001$). However, the cluster identified in the southwest region of the state, dominated by lineage IIb, through multinomial spatial scan statistics moved somewhat northward and decreased in size without the secondary cases.

Reporting Bias

We assessed potential reporting bias by county. Reporting of patients who have tested positive is considered near 100% (*11*), but testing intensity may vary by provider. *E. coli* O157:H7 is most often detected by fecal specimen culture, a test that also detects *Campylobacter*, *Salmonella*, and *Shigella*. If providers in an area have heightened awareness of *E. coli* O157:H7 and are more likely to test for it than in other areas, we would expect that detection of these other pathogens would also be higher. There is overlap in the epidemiology of *E. coli* O157:H7, *Campylobacter*, and *Salmonella*, so some correlation is expected. However, risk factors for *Shigella* are generally different (*12*). If there were reporting bias, we would expect this to have the greatest impact on the observed incidence of milder *E. coli* O157:H7 strains.

Case counts by county for 2005–2014 for campylobacteriosis, salmonellosis, and shigellosis were obtained from the Washington State Communicable Disease Reports for 2009 and 2014 (each contained 5 years of data) (*13,14*). We calculated incidence rates using county populations as reported in 2010 U.S. Census TIGER/Line Shapefiles (*15*). Using the GISTools (*16*) package in R, we mapped the incidence quintile of each of the 4 pathogens at the county level for the study period to assess the potential for reporting bias (Technical Appendix Figure 4). Two counties, Yakima and Grant, appear in the uppermost quintile of incidence for each of the 4 diseases. However, incidence of rare lineage *E. coli* O157:H7 in this region is remarkably low (main article Figure 1; Technical Appendix Figure 2). Infections caused by these bacteria are generally milder (main article Table) and would be the type whose numbers would be exaggerated in the presence of heightened testing. Thus, it is unlikely that reporting bias is responsible for the observed results.

Data

Genomic data, with limited metadata, on all isolates used in the study are provided in Technical Appendix 2 (https://wwwnc.cdc.gov/EID/article/23/1/17-0851-Techapp2.xlsx). These include genomic data on all 1,160 *E. coli* O157:H7 isolates from reported, culture-confirmed

cases in Washington state, 2005–2014. Phylogenetic lineage was determined directly using the 48-plex SNP assay developed by Jung et al. (*17*) or was inferred from a typed isolate with the same PFGE profile. Shiga toxin bacteriophage insertion typing and typing for clade according to the method used by Manning et al. (*2*) were conducted on only a subset of isolates. NT, not typed; PFGE, pulsed field gel electrophoresis; SBI, Shiga toxin bacteriophage insertion typing; SDM, Shannon Manning clade/genotype.

References

1. Iyoda S, Manning SD, Seto K, Kimata K, Isobe J, Etoh Y, et al. Phylogenetic clades 6 and 8 of enterohemorrhagic *Escherichia coli* O157:H7 with particular stx subtypes are more frequently found in isolates from hemolytic uremic syndrome patients than from asymptomatic carriers. Open Forum Infect Dis. 2014;1:ofu061. http://dx.doi.org/10.1093/ofid/ofu061

2. Manning SD, Motiwala AS, Springman AC, Qi W, Lacher DW, Ouellette LM, et al. Variation in virulence among clades of *Escherichia coli* O157:H7 associated with disease outbreaks. Proc Natl Acad Sci U S A. 2008;105:4868–73. http://dx.doi.org/10.1073/pnas.0710834105

3. Pianciola L, Chinen I, Mazzeo M, Miliwebsky E, González G, Müller C, et al. Genotypic characterization of *Escherichia coli* O157:H7 strains that cause diarrhea and hemolytic uremic syndrome in Neuquén, Argentina. Int J Med Microbiol. 2014;304:499–504. http://dx.doi.org/10.1016/j.ijmm.2014.02.011

4. Diggle PJ, Zheng P, Durr P. Nonparametric estimation of spatial segregation in a multivariate point process: bovine tuberculosis in Cornwall, UK. Appl Stat. 2005;54:645–58. http://dx.doi.org/10.1111/j.1467-9876.2005.05373.x

5. R Core Team. R: a language and environment for statistical computing. Vienna: R Foundation for Statistical Computing; 2015.

6. Zheng P, Diggle PJ. Spatialkernel: nonparametric estimation of spatial segregation in a multivariate point process; R package version 0.4-19. 2013 [cited 2015 May 27]. https://CRAN.R-project.org/package=spatialkernel

7. Dixon PM. Nearest-neighbor contingency table analysis of spatial segregation for several species. Écoscience. 2002;9:142–51. http://dx.doi.org/10.1080/11956860.2002.11682700

8. de la Cruz M. Metodos Para Analizar Datos Puntuales. In: Maestre, FT, Escudero, A, Bonet, A, editors. Introduccion Al Analisis Espacial De Datos En Ecologia Y Ciencias Ambientales: Metodos Y Aplicaciones. Madrid: Asociacion Espanola de Ecologia Terrestre, Universidad Rey Juan Carlos and Caja de Ahorros del Mediterraneo; 2008. p. 76–127.

9. Jung I, Kulldorff M, Richard OJ. A spatial scan statistic for multinomial data. Stat Med. 2010;29:1910–8. http://dx.doi.org/10.1002/sim.3951

10. Kulldorff M, Information Management Services I. Satscan^{tm} V8.0: Software for the spatial and space-time scan statistics. 2009 [cited 2015 May 27]. http://www.satscan.org/

11. Scallan E, Hoekstra RM, Angulo FJ, Tauxe RV, Widdowson MA, Roy SL, et al. Foodborne illness acquired in the United States—major pathogens. Emerg Infect Dis. 2011;17:7–15. http://dx.doi.org/10.3201/eid1701.P11101

12. Denno DM, Keene WE, Hutter CM, Koepsell JK, Patnode M, Flodin-Hursh D, et al. Tri-county comprehensive assessment of risk factors for sporadic reportable bacterial enteric infection in children. J Infect Dis. 2009;199:467–76. http://dx.doi.org/10.1086/596555

13. Communicable Disease Epidemiology Section. Communicable Disease Report 2014. Shoreline, WA: Washington State Department of Health; 2014. p. 95.

14. Communicable Disease Epidemiology Section. Communicable Disease Report 2009. Shoreline, WA: Washington State Department of Health; 2009. p. 91.

15. United States Census Bureau. 2012 Tiger/Line Shapefiles (Machine-Readable Data Files). Washington, DC: The Bureau; 2012.

16. Brundsdon C, Chen H. GIStools: some further GIS capabilities for R; R package version 0.7-4. 2014 [cited 2015 May 27]. https://CRAN.R-project.org/package=GISTools

17. Jung WK, Bono JL, Clawson ML, Leopold SR, Shringi S, Besser TE. Lineage and genogroup-defining single nucleotide polymorphisms of *Escherichia coli* O157:H7. Appl Environ Microbiol. 2013;79:7036–41. http://dx.doi.org/10.1128/AEM.02173-13

Technical Appendix Table 1. Association of known risk factors with phylogenetic lineage*

Variable	Statewide frequency	Statewide OR (95% CI)	Southwest region (n = 234) OR (95% CI)	Northwest region (n = 289) OR (95% CI)	South-central region (n = 109) OR (95% CI)
Hispanic ethnicity (vs. non-Hispanic)					
Lineage Ib	46/372	Ref	Ref	Ref	Ref
Lineage IIa	32/197	1.13 (0.67, 1.91)	0.3 (0.03, 2.86)	2.79 (0.66, 11.83)	0.87 (0.33, 2.25)
Lineage IIb	19/152	1.13 (0.61, 2.11)	0.99 (0.3, 3.33)	3.24 (0.62, 16.86)	0.73 (0.12, 4.37)
Rare lineage	6/42	1.21 (0.46, 3.15)	8.15 (0.89, 75.06)	1.98 (0.18, 21.31)	0 (0, Inf)†
American Indian (vs. white race)‡					
Lineage Ib	5/377	Ref	Ref	Ref	Ref
Lineage IIa	7/196	**3.82 (1.13, 12.95)§**	NA	NA	NA
Lineage IIb	0/148	0 (0, Inf)†	NA	NA	NA
Rare lineage	0/40	0 (0, Inf)†	NA	NA	NA
Asian race (vs. white race)‡					
Lineage Ib	24/377	Ref	Ref	Ref	Ref
Lineage IIa	7/196	0.53 (0.22, 1.28)	NA	NA	NA
Lineage IIb	19/148	**2.03 (1.02, 4.01)§**	NA	NA	NA
Rare lineage	2/40	0.72 (0.16, 3.22)	NA	NA	NA
Black race (vs. white race)‡					
Lineage Ib	12/377	Ref	Ref	Ref	Ref
Lineage IIa	5/196	0.81 (0.27, 2.43)	NA	NA	NA
Lineage IIb	5/148	1.02 (0.34, 3.06)	NA	NA	NA
Rare lineage	0/40	0 (0, Inf)†	NA	NA	NA
Other/multiple race (vs. white race)‡					
Lineage Ib	16/377	Ref	Ref	Ref	Ref
Lineage IIa	9/196	0.94 (0.39, 2.23)	NA	NA	NA
Lineage IIb	11/148	1.59 (0.69, 3.68)	NA	NA	NA
Rare lineage	1/40	0.55 (0.07, 4.32)	NA	NA	NA
Contact with a laboratory-confirmed case					
Lineage Ib	59/531	Ref	Ref	Ref	Ref
Lineage IIa	39/228	1.34 (0.84, 2.15)	0.88 (0.3, 2.6)	1.48 (0.63, 3.49)	0.99 (0.25, 3.96)
Lineage IIb	43/176	**1.96 (1.21, 3.16)¶**	**2.7 (1.15, 6.31)§**	2.03 (0.78, 5.25)	2.74 (0.44, 17.21)
Rare lineage	3/60	0.41 (0.12, 1.37)	0.42 (0.05, 3.82)	0.39 (0.05, 3.24)	0 (0, Inf)†
Epidemiologic link to a confirmed or probable case					
Lineage Ib	74/522	Ref	Ref	Ref	Ref
Lineage IIa	41/221	1.25 (0.80, 1.96)	1.07 (0.37, 3.05)	0.97 (0.42, 2.25)	0.99 (0.24, 3.98)
Lineage IIb	51/172	**1.94 (1.24, 3.03)¶**	2.17 (0.94, 4.98)	1.41 (0.56, 3.55)	4.72 (0.85, 26.07)
Rare lineage	3/60	0.32 (0.10, 1.06)	0.33 (0.04, 2.95)	0.29 (0.04, 2.39)	0 (0, Inf)†
Underlying illness					
Lineage Ib	66/530	Ref	Ref	Ref	Ref
Lineage IIa	27/233	1.20 (0.70, 2.06)	2.87 (0.86, 9.61)	0.83 (0.2, 3.37)	4.07 (0.5, 33.02)
Lineage IIb	19/184	1.11 (0.61, 2.01)	1.17 (0.36, 3.77)	0.73 (0.15, 3.59)	6.07 (0.33, 111.66)
Rare lineage	2/62	**0.19 (0.04, 0.85)§**	0.59 (0.06, 5.84)	0.42 (0.05, 3.73)	0 (0, Inf)†
Contact with diapered or incontinent child or adult					
Lineage Ib	122/545	Ref	Ref	Ref	Ref
Lineage IIa	65/231	1.10 (0.75, 1.61)	0.91 (0.37, 2.22)	0.94 (0.42, 2.1)	1.43 (0.54, 3.79)
Lineage IIb	60/187	1.28 (0.86, 1.91)	1.57 (0.76, 3.26)	1.58 (0.67, 3.73)	0.82 (0.13, 5.17)
Rare lineage	8/62	0.53 (0.24, 1.16)	1.44 (0.36, 5.72)	0.19 (0.02, 1.52)	0.69 (0.06, 7.7)
Attends childcare or preschool					
Lineage Ib	39/523	Ref	Ref	Ref	Ref
Lineage IIa	22/235	DNC	2.7 (0.68, 10.64)	1.7 (0.42, 6.86)	1.19 (0.21, 6.56)
Lineage IIb	27/181	DNC	**3.17 (1.03, 9.7)§**	2.16 (0.55, 8.57)	0 (0, Inf)†
Rare lineage	0/59	DNC	0 (0, Inf)†	0 (0, Inf)†	0 (0, Inf)†
Employed as a healthcare worker					
Lineage Ib	17/525	Ref	Ref	Ref	Ref
Lineage IIa	8/232	DNC	3.06 (0.44, 21.55)	0.7 (0.06, 8.42)	0 (0, Inf)†
Lineage IIb	7/182	DNC	0.71 (0.06, 8.23)	1.52 (0.15, 15.38)	2.41 (0.18, 33.1)
Rare lineage	1/62	DNC	0 (0, Inf)†	0 (0, Inf)†	0 (0, Inf)†
Employed as a food worker					
Lineage Ib	18/539	Ref	Ref	Ref	Ref
Lineage IIa	12/244	1.64 (0.74, 3.59)	1.4 (0.1, 19.6)	1.58 (0.45, 5.56)	0 (0, Inf)†
Lineage IIb	4/188	0.74 (0.24, 2.28)	1.61 (0.21, 12.62)	0.53 (0.06, 4.44)	0 (0, Inf)†
Rare lineage	2/60	0.99 (0.22, 4.41)	0 (0, Inf)†	1.11 (0.13, 9.77)	0 (0, Inf)†

Variable	Statewide frequency	Statewide OR (95% CI)	Southwest region (n = 234) OR (95% CI)	Northwest region (n = 289) OR (95% CI)	South-central region (n = 109) OR (95% CI)
Works with animals or animal products					
Lineage Ib	24/524	Ref	Ref	Ref	Ref
Lineage IIa	5/196	0.46 (0.16, 1.27)	0 (0, Inf)†	0.31 (0.04, 2.57)	0.87 (0.12, 6.08)
Lineage IIb	5/163	0.84 (0.30, 2.40)	0.77 (0.11, 5.45)	0 (0, Inf)†	2.17 (0.19, 24.56)
Rare lineage	3/53	1.14 (0.33, 4.00)	2.87 (0.25, 33.45)	1.73 (0.32, 9.22)	0 (0, Inf)†
Any contact with animals					
Lineage Ib	300/521	Ref	Ref	Ref	Ref
Lineage IIa	115/200	0.81 (0.57, 1.15)	0.84 (0.34, 2.09)	0.56 (0.26, 1.24)	0.48 (0.18, 1.3)
Lineage IIb	90/167	0.78 (0.54, 1.14)	1.9 (0.89, 4.06)	0.48 (0.2, 1.15)	**0.16 (0.03, 0.88)§**
Rare lineage	27/52	0.8 (0.44, 1.45)	Inf (0, Inf)†	0.73 (0.24, 2.26)	0.3 (0.02, 3.63)
Contact with cattle, cows, or calves					
Lineage Ib	63/471	Ref	Ref	Ref	Ref
Lineage IIa	30/188	1.06 (0.64, 1.78)	1.11 (0.32, 3.81)	0.68 (0.26, 1.78)	1.06 (0.36, 3.12)
Lineage IIb	13/151	0.59 (0.3, 1.14)	1.04 (0.38, 2.84)	0.14 (0.02, 1.07)	0 (0, Inf)†
Rare lineage	7/49	1.19 (0.5, 2.81)	0.95 (0.1, 8.81)	0.92 (0.24, 3.54)	0 (0, Inf)†
Case or household member lives or works on a farm or dairy					
Lineage Ib	67/526	Ref	Ref	Ref	Ref
Lineage IIa	24/191	0.86 (0.50, 1.46)	0.47 (0.09, 2.5)	0.96 (0.37, 2.44)	1.06 (0.36, 3.13)
Lineage IIb	15/169	0.67 (0.35, 1.27)	1.62 (0.58, 4.48)	0 (0, Inf)†	0.33 (0.04, 2.95)
Rare lineage	7/53	1.08 (0.47, 2.52)	1.35 (0.14, 13)	0.99 (0.26, 3.82)	1.39 (0.11, 17.56)
Visited a zoo, farm, fair, or pet shop					
Lineage Ib	99/526	Ref	Ref	Ref	Ref
Lineage IIa	49/200	1.31 (0.86, 2)	1.59 (0.61, 4.17)	0.93 (0.41, 2.12)	1 (0.28, 3.53)
Lineage IIb	25/166	**0.59 (0.35, 1)§**	0.88 (0.4, 1.94)	**0.17 (0.04, 0.78)§**	0 (0, Inf)†
Rare lineage	11/53	1.11 (0.53, 2.33)	0.52 (0.06, 4.65)	0.6 (0.16, 2.32)	2.65 (0.2, 34.74)
Recreational water exposure					
Lineage Ib	130/548	Ref	Ref	Ref	Ref
Lineage IIa	57/229	0.96 (0.65, 1.41)	0.51 (0.18, 1.45)	0.53 (0.24, 1.17)	0.79 (0.25, 2.56)
Lineage IIb	38/174	0.82 (0.53, 1.27)	**0.38 (0.16, 0.93)§**	0.44 (0.16, 1.24)	**6.39 (1.09, 37.47)§**
Rare lineage	12/60	0.79 (0.40, 1.57)	0.66 (0.13, 3.41)	0.77 (0.22, 2.73)	1.41 (0.12, 16.12)
Drank untreated/unchlorinated water					
Lineage Ib	61/531	Ref	Ref	Ref	Ref
Lineage IIa	29/219	0.96 (0.58, 1.57)	**4.49 (1.48, 13.57)¶**	0.89 (0.27, 2.87)	**0.16 (0.04, 0.63)¶**
Lineage IIb	26/169	1.27 (0.74, 2.16)	**3.76 (1.38, 10.28)¶**	1.5 (0.44, 5.15)	0.27 (0.03, 2.38)
Rare lineage	7/53	1.14 (0.49, 2.66)	1.68 (0.29, 9.69)	2.14 (0.41, 11.07)	0 (0, Inf)†
Well is source of drinking water					
Lineage Ib	136/559	Ref	Ref	Ref	Ref
Lineage IIa	59/236	0.91 (0.62, 1.32)	1.1 (0.47, 2.54)	1.1 (0.5, 2.41)	0.47 (0.19, 1.17)
Lineage IIb	35/186	0.77 (0.48, 1.21)	1.06 (0.52, 2.12)	0.7 (0.24, 2)	**0.08 (0.01, 0.72)§**
Rare lineage	14/62	0.87 (0.46, 1.65)	0.49 (0.11, 2.09)	1.13 (0.33, 3.84)	0.18 (0.02, 1.73)
Consumed food from a restaurant					
Lineage Ib	384/505	Ref	Ref	Ref	Ref
Lineage IIa	166/216	1.22 (0.81, 1.83)	1.82 (0.69, 4.81)	0.93 (0.4, 2.17)	0.66 (0.25, 1.72)
Lineage IIb	132/171	1.09 (0.7, 1.68)	1.09 (0.5, 2.39)	0.72 (0.29, 1.78)	Inf (0, Inf)†
Rare lineage	43/54	1.23 (0.61, 2.49)	0.74 (0.19, 2.82)	0.82 (0.24, 2.79)	1.61 (0.15, 17.53)
Consumed food from a group meal					
Lineage Ib	144/531	Ref	Ref	Ref	Ref
Lineage IIa	65/227	1.1 (0.77, 1.59)	0.53 (0.19, 1.48)	1.56 (0.72, 3.39)	0.73 (0.28, 1.92)
Lineage IIb	59/179	1.24 (0.84, 1.82)	1.18 (0.58, 2.4)	**2.45 (1.06, 5.71)§**	0.27 (0.03, 2.52)
Rare lineage	17/58	1.16 (0.64, 2.13)	0.58 (0.12, 2.86)	**3.1 (1.02, 9.4)§**	0.46 (0.04, 4.8)
Handled raw meat					
Lineage Ib	122/542	Ref	Ref	Ref	Ref
Lineage IIa	43/226	0.86 (0.54, 1.38)	1.21 (0.4, 3.64)	0.75 (0.3, 1.88)	1.5 (0.37, 6.14)
Lineage IIb	31/182	0.92 (0.55, 1.53)	1.41 (0.55, 3.61)	0.23 (0.05, 1.08)	0.51 (0.07, 3.9)
Rare lineage	15/62	1.09 (0.54, 2.17)	1.47 (0.33, 6.47)	0.62 (0.15, 2.49)	2.14 (0.17, 27.6)
Consumed meat					
Lineage Ib	314/521	Ref	Ref	Ref	Ref
Lineage IIa	138/223	1.09 (0.77, 1.53)	1.09 (0.48, 2.47)	1.33 (0.59, 2.99)	1.45 (0.58, 3.62)
Lineage IIb	106/175	1.07 (0.74, 1.55)	1.25 (0.64, 2.44)	1.83 (0.63, 5.37)	1.3 (0.28, 6.08)
Rare lineage	31/56	0.75 (0.43, 1.33)	0.59 (0.16, 2.13)	0.46 (0.15, 1.42)	0.77 (0.11, 5.23)
Consumed ground beef					
Lineage Ib	331/539	Ref	Ref	Ref	Ref
Lineage IIa	132/229	0.85 (0.61, 1.18)	0.94 (0.39, 2.3)	0.88 (0.43, 1.8)	0.82 (0.32, 2.09)
Lineage IIb	103/180	0.85 (0.59, 1.22)	0.87 (0.43, 1.76)	**0.27 (0.11, 0.65)¶**	0.82 (0.18, 3.78)
Rare lineage	31/57	0.76 (0.44, 1.34)	1.52 (0.29, 8.01)	0.6 (0.22, 1.69)	0.31 (0.04, 2.14)
Consumed intact beef					
Lineage Ib	283/462	Ref	Ref	Ref	Ref

Variable	Statewide frequency	Statewide OR (95% CI)	Southwest region (n = 234) OR (95% CI)	Northwest region (n = 289) OR (95% CI)	South-central region (n = 109) OR (95% CI)
Lineage IIa	116/185	1.07 (0.74, 1.56)	0.55 (0.2, 1.5)	0.87 (0.4, 1.89)	2.81 (0.85, 9.3)
Lineage IIb	90/156	0.86 (0.58, 1.28)	0.96 (0.42, 2.17)	**0.35 (0.14, 0.87)§**	1.36 (0.22, 8.52)
Rare lineage	29/46	1.17 (0.61, 2.27)	2.77 (0.3, 25.43)	1.54 (0.43, 5.51)	0.31 (0.01, 7.79)
Consumed venison or other wild game meat					
Lineage Ib	15/521	Ref	Ref	Ref	Ref
Lineage IIa	3/195	0.37 (0.08, 1.68)	0 (0, Inf)†	0 (0, Inf)†	0 (0, Inf)†
Lineage IIb	10/169	1.97 (0.81, 4.79)	1.35 (0.4, 4.58)	1.22 (0.13, 11.12)	0 (0, Inf)†
Rare lineage	5/53	**3.56 (1.23, 10.32)§**	1.56 (0.16, 14.98)	3.56 (0.58, 21.96)	**34.96 (1.03, 1187.37)§**
Consumed raw milk					
Lineage Ib	16/551	Ref	Ref	Ref	Ref
Lineage IIa	6/232	0.82 (0.3, 2.23)	4.04 (0.22, 75.92)	0.38 (0.04, 3.72)	0 (0, Inf)†
Lineage IIb	18/183	**2.46 (1.15, 5.28)§**	**17.33 (2.05, 146.5)¶**	0 (0, Inf)†	24.32 (0.81, 726.95)
Rare lineage	1/60	0.63 (0.08, 4.88)	0 (0, Inf)†	0 (0, Inf)†	0 (0, Inf)†
Consumed unpasteurized juice					
Lineage Ib	11/496	Ref	Ref	Ref	Ref
Lineage IIa	3/219	0.34 (0.09, 1.27)	0.8 (0.11, 6.04)	0 (0, Inf)†	0 (0, Inf)†
Lineage IIb	7/163	1.53 (0.55, 4.29)	0.6 (0.09, 4.03)	7.08 (0.37, 137.1)	5.9 (0.35, 100.4)
Rare lineage	3/55	2.31 (0.61, 8.78)	2.39 (0.21, 27.47)	**23.08 (1.52, 351.69)§**	0 (0, Inf)†
Consumed raw fruits or vegetables					
Lineage Ib	435/514	Ref	Ref	Ref	Ref
Lineage IIa	184/205	**1.81 (1.05, 3.11)§**	6.88 (0.84, 56.67)	2.55 (0.52, 12.41)	1.34 (0.43, 4.16)
Lineage IIb	144/170	1.25 (0.74, 2.1)	1.51 (0.62, 3.64)	0.78 (0.23, 2.6)	1.97 (0.2, 19.15)
Rare lineage	43/48	1.5 (0.57, 4)	Inf (0, Inf)†	2.11 (0.25, 17.82)	0.37 (0.02, 5.85)
Consumed sprouts					
Lineage Ib	22/537	Ref	Ref	Ref	Ref
Lineage IIa	12/231	1.45 (0.68, 3.11)	1.87 (0.23, 15.21)	2.98 (0.57, 15.62)	Inf (0, Inf)†
Lineage IIb	12/180	2 (0.94, 4.27)	1.11 (0.17, 7.45)	**5.17 (1.04, 25.74)§**	0.5 (0, Inf)
Rare lineage	4/57	1.94 (0.64, 5.94)	0 (0, Inf)†	**7.32 (1.11, 48.28)§**	0.24 (0, Inf)
Consumed fresh herbs					
Lineage Ib	102/524	Ref	Ref	Ref	Ref
Lineage IIa	44/216	0.83 (0.54, 1.27)	0.95 (0.32, 2.79)	0.88 (0.37, 2.1)	**0.19 (0.04, 0.77)§**
Lineage IIb	35/178	1.01 (0.64, 1.6)	0.78 (0.29, 2.13)	1.51 (0.59, 3.85)	0.39 (0.04, 3.57)
Rare lineage	9/56	0.7 (0.32, 1.55)	0 (0, Inf)†	1.11 (0.29, 4.3)	0.39 (0.03, 4.47)
Traveled outside the state, the country, or usual routine					
Lineage Ib	143/571	Ref	Ref	Ref	Ref
Lineage IIa	52/246	0.78 (0.53, 1.13)	0.45 (0.17, 1.19)	0.37 (0.14, 1)	1.09 (0.34, 3.54)
Lineage IIb	54/197	1.08 (0.74, 1.59)	0.86 (0.44, 1.7)	1.71 (0.73, 4)	1.53 (0.26, 9.01)
Rare lineage	26/64	**2.03 (1.17, 3.50)§**	0.66 (0.16, 2.65)	**3.72 (1.27, 10.87)§**	**7.45 (1.03, 54.07)§**

*All analyses are multinomial logistic regression, using lineage Ib as the reference group, adjusted for age, sex, and year. The statewide analysis was conducted using a generalized additive model to additionally adjust for latitude and longitude using a thin plate spline bivariate smooth. Statistically significant results are shown in bold text. "Rare lineage" includes 12 different clinically rare lineages. CI, confidence interval; DNC, did not converge; Inf, infinity; NA, not applicable; OR, odds ratio; Ref, reference

†Odds ratios of 0 are reported where 0 cases of the lineage under analysis existed in the category. Odds ratios of infinity are reported where 0 cases of the reference lineage (Ib) existed in the category. Confidence intervals were not estimated for these ORs, indicated by (0, Inf).

‡ Analyses marked NA could not be performed or were considered unreliable because of sparse data in these categories. Not all models converged because of sparse data in some categories.

§ $p < 0.05$

¶ $p < 0.01$

Technical Appendix Table 2. Multinomial generalized additive model sensitivity analysis

Model	Latitude/longitude p-value	AIC
Bivariate thin plate regression spline model for latitude/longitude, age, and sex covariates*	IIa: 0.127<br>IIb: <0.001<br>Rare: 0.692	1337
Intercept only	NA	1396
Univariate thin plate regression spline models for latitude and longitude	IIa latitude: 0.022<br>IIa longitude: 0.967<br>IIb latitude: <0.001<br>IIb longitude: <0.001<br>Rare latitude: 0.399<br>Rare longitude: 0.734	1338
Bivariate thin plate regression spline model for latitude/longitude	IIa: 0.071<br>IIb: <0.001<br>Rare: 0.688	1340
Bivariate thin plate regression spline model for latitude/longitude, age and sex covariates, basis dimension doubled	IIa: 0.127<br>IIb: <0.001<br>Rare: 0.691	1336
Cubic regression spline models for latitude and longitude, age and sex covariates	IIa latitude: 0.042<br>IIa longitude: 0.845<br>IIb latitude: <0.001<br>IIb longitude: <0.001<br>Rare latitude: 0.425<br>Rare longitude: 0.646	1336
Bivariate tensor product spline model for latitude/longitude, age and sex covariates	IIa: 0.077<br>IIb: <0.001<br>Rare: 0.860	1338
Bivariate thin plate regression spline model for latitude/longitude, age and sex covariates, using lineage IIa as the comparator instead of Ib	Ib: 0.127<br>IIb: <0.001<br>Rare: 0.189	1969

Model	Latitude/longitude p-value	AIC
Bivariate thin plate regression spline model for latitude/longitude; age, sex, and year covariates	IIa: 0.104<br>IIb: <0.001<br>Rare: 0.739	1273
Thin plate regression spline models for latitude/longitude (bivariate) and year (univariate), age and sex covariates	IIa: 0.116<br>IIb: <0.001<br>Rare: 0.730	1237
Trivariate thin plate regression spline model for latitude/longitude/year, age and sex covariates	IIa latitude/longitude/year: <0.001<br>IIb latitude/longitude/year: <0.001<br>Rare latitude/longitude/year: 0.475	1174

*Primary model. AIC, Akaike information criterion; NA, not applicable

Technical Appendix Table 3. Dixon nearest-neighbor contingency table analysis of spatial segregation

Lineage	df*	χ^2	p-value
Overall	12	96.19	<0.001
Ib	3	8.02	0.046
IIa	3	15.08	0.002
IIb	3	75.61	<0.001
Rare	3	4.04	0.257

* df, degrees of freedom

Technical Appendix Table 4. Pairwise segregation of lineages using Dixon’s nearest-neighbor contingency table method

From	To	Observed Count	Expected Count	*S*	*Z*-score	p-value
Ib	Ib	343	308.84	0.10	2.61	0.009
Ib	IIa	115	137.26	–0.10	–2.19	0.028
Ib	IIb	92	105.06	–0.07	–1.44	0.150
Ib	Rare	36	34.84	0.02	0.21	0.832
IIa	Ib	122	137.26	–0.10	–1.80	0.072
IIa	IIa	90	60.67	0.24	3.61	<0.001
IIa	IIb	40	46.61	–0.08	–1.08	0.280
IIa	Rare	8	15.46	–0.30	–2.00	0.046
IIb	Ib	80	105.06	–0.22	–3.42	<0.001
IIb	IIa	24	46.61	–0.35	–3.80	<0.001
IIb	IIb	91	35.50	0.59	8.50	<0.001
IIb	Rare	4	11.83	–0.49	–2.39	0.017
Rare	Ib	43	34.84	0.22	1.98	0.047
Rare	IIa	11	15.46	–0.18	–1.30	0.195
Rare	IIb	9	11.83	–0.14	–0.91	0.362
Rare	Rare	3	3.86	–0.12	–0.36	0.717

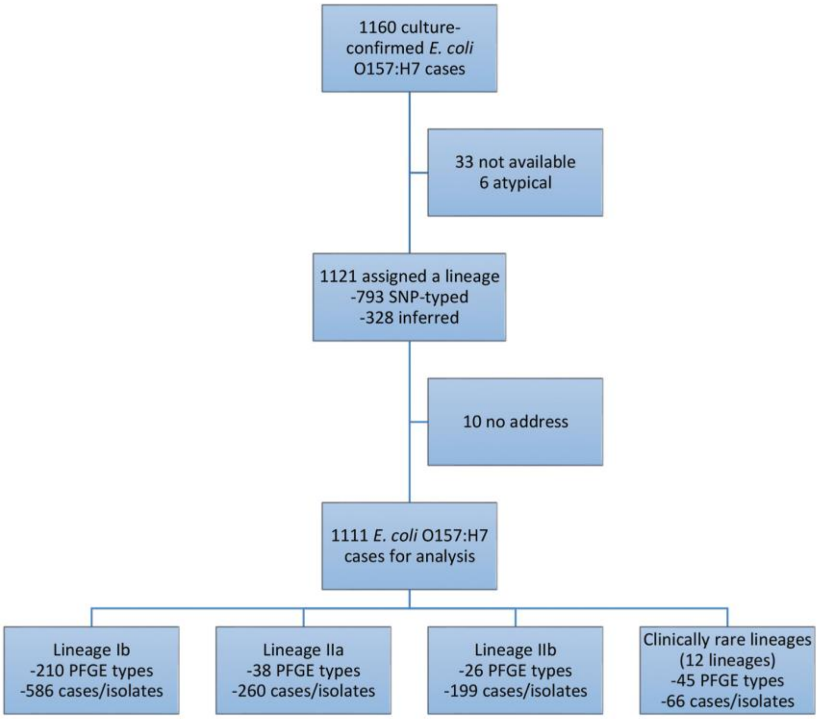

Technical Appendix Figure 1. Of the 1,160 culture-confirmed *E. coli* O157:H7 cases reported in Washington state during 2005–2014, 1,111 were included in the analysis. Isolates from these 1,111 cases spanned 15 phylogenetic lineages using the 48-plex single nucleotide polymorphism assay developed by Jung et al. (*17*). Three lineages, Ib, IIa, and IIb, constituted 94% of isolates. Isolates from the remaining 12 lineages were grouped into a “clinically rare” group. XbaI pulsed field gel electrophoresis (PFGE) types were determined, and all isolates of a given PFGE type belonged to the same phylogenetic lineage. The number of PFGE types and case isolates belonging to each lineage are shown.

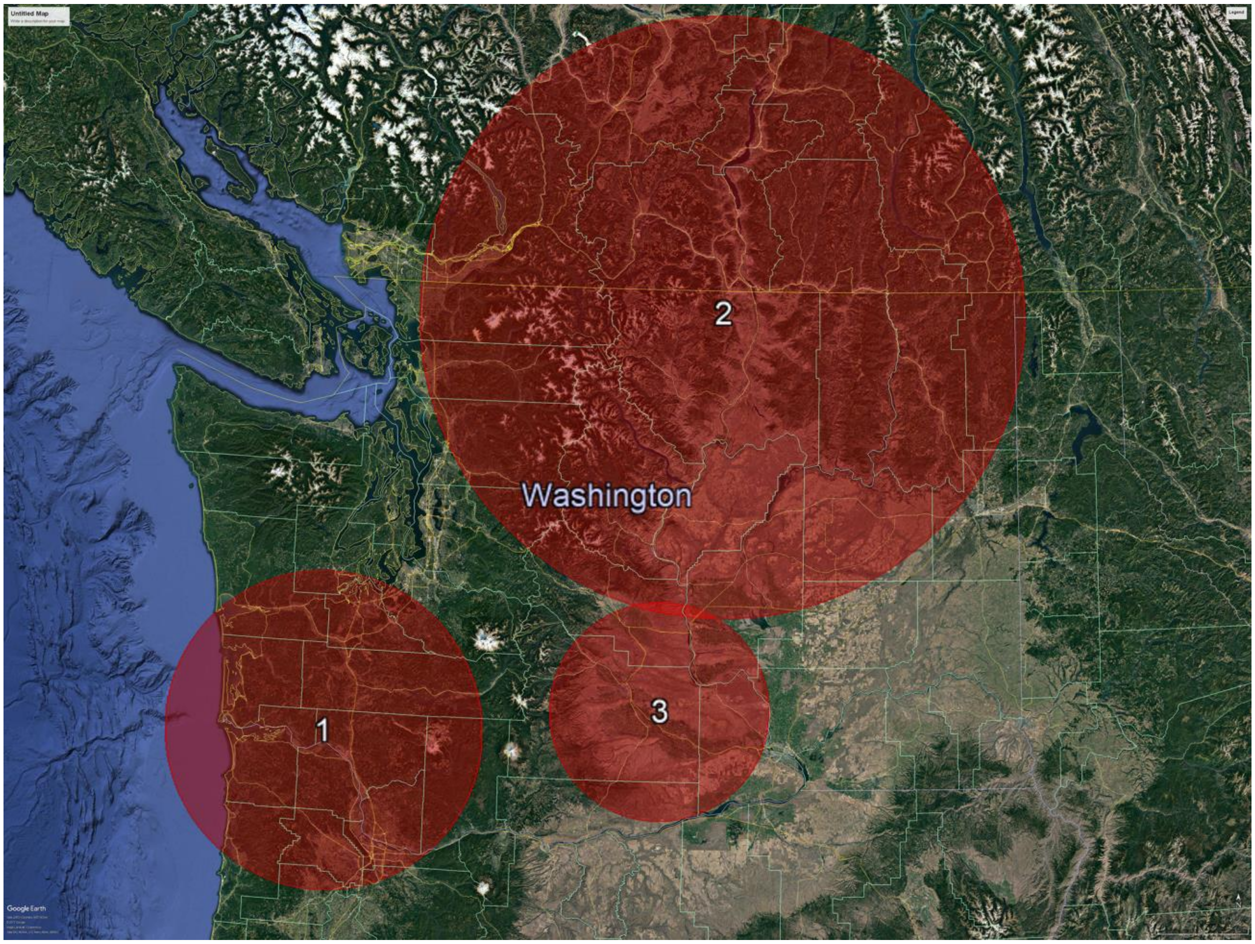

Technical Appendix Figure 2. Statistically significant clusters of variant phylogenetic lineage. Multinomial spatial scan statistics were used to identify clusters in which the distribution of lineages varied from that of the rest of the state. Clusters were restricted to a maximum of 20% of cases. Cluster 1: 203 cases; Ib relative risk (RR) = 0.66, IIa RR = 0.94, IIb RR = 2.59, Rare RR = 0.80; p = 0.001. Cluster 2: 185 cases; Ib RR = 1.37, IIa RR = 0.65, IIb RR = 0.29, Rare RR = 1.88; p = 0.001. Cluster 3: 79 cases; Ib RR = 1.14, IIa RR = 1.70, IIb RR = 0.13, Rare RR = 0; p = 0.006.

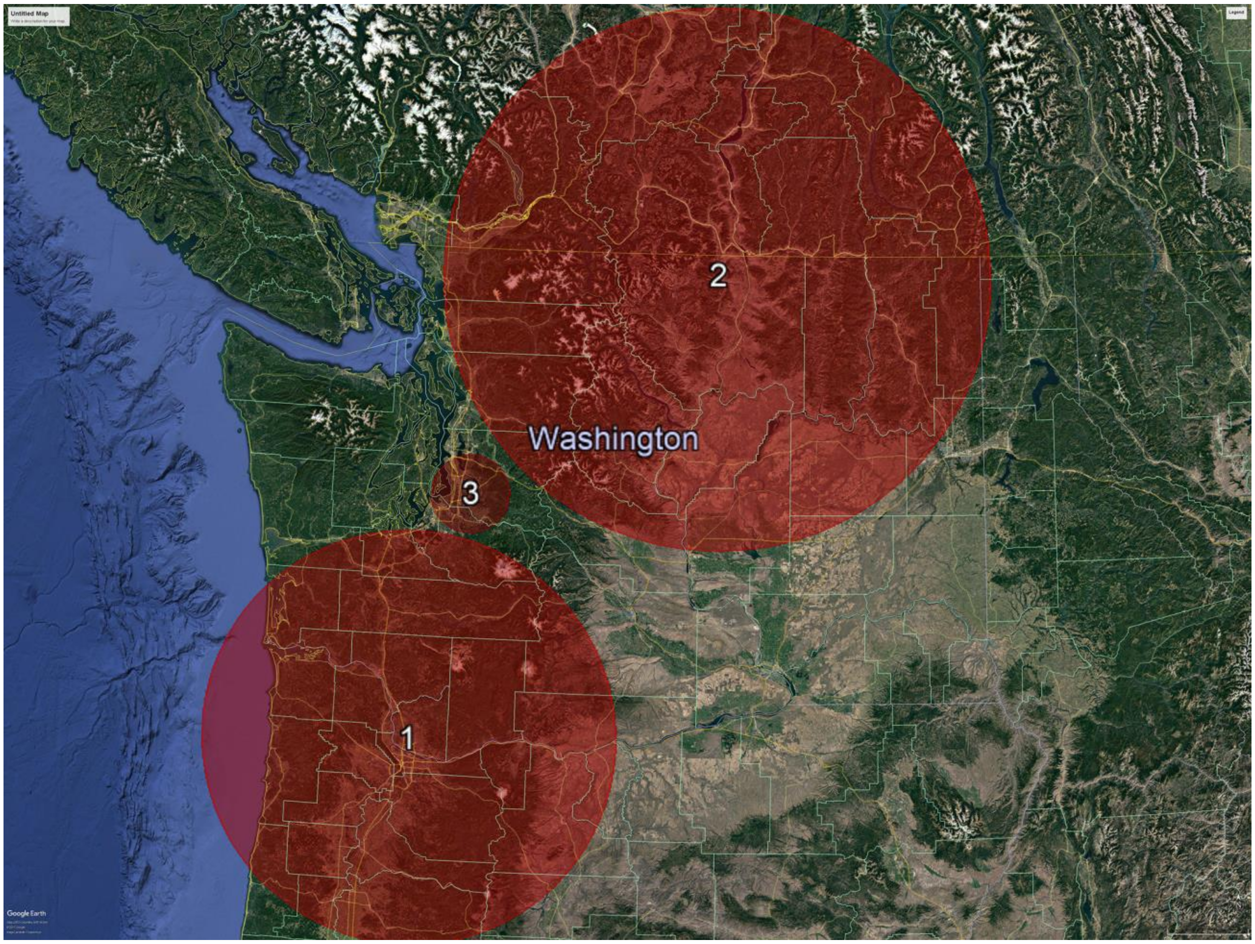

Technical Appendix Figure 3. Statistically significant space-time clusters of variant phylogenetic lineage. Multinomial spatiotemporal scan statistics were used to identify clusters in which the distribution of lineages varied from that of the rest of the state during years outside the cluster. Clusters were restricted to a maximum of 20% of cases and 50% of the study window. Cluster 1: 2009–2012; 76 cases; Ib relative risk (RR) = 0.28, IIa RR = 0.49, IIb RR = 4.45, Rare RR = 1.36; p = 0.001. Cluster 2: 2005–2009; 107 cases; Ib RR = 1.61, IIa RR = 0.22, IIb RR = 0.19, Rare RR = 1.88; p = 0.001. Cluster 3: 2009–2010; 46 cases; Ib RR = 0.65, IIa RR = 0.09, IIb RR = 3.63, Rare RR = 0.72; p = 0.002.

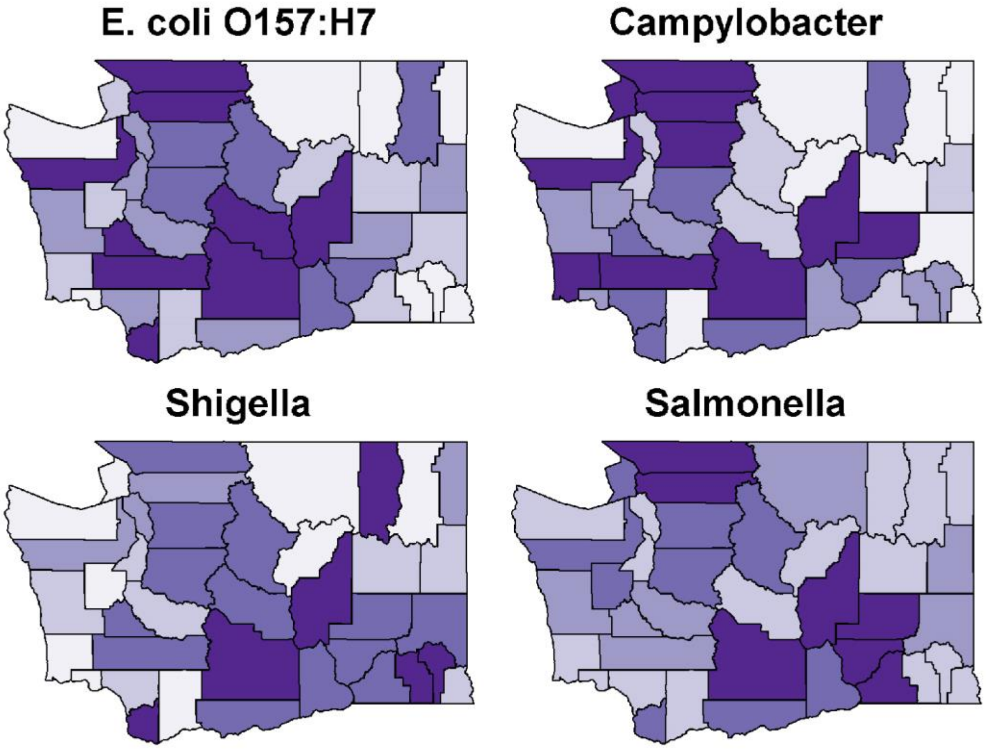

Technical Appendix Figure 4. Incidence rate quintiles by county of reported *E. coli* O157, *Campylobacter*, *Shigella,* and *Salmonella*, 2005–2014. Tests are routinely performed for these 4 pathogens simultaneously, and uniformly high rates may suggest higher testing intensity in a county.
